# Antimicrobial Responses to Bacterial Metabolic Activity and Biofilm Formation Studied Using Microbial Fuel Cell-Based Biosensors

**DOI:** 10.3390/bios14120606

**Published:** 2024-12-11

**Authors:** Wenguo Wu, Huiya Hong, Jia Lin, Dayun Yang

**Affiliations:** 1 College of Chemical Engineering, Huaqiao University, Xiamen 361021, China; 22013087007@stu.hqu.edu.cn (H.H.); linjia3456789@163.com (J.L.); 2 Fujian Key Laboratory of Translational Research in Cancer and Neurodegenerative Diseases, School of Basic Medical Sciences, Fujian Medical University, Fuzhou 350108, China

**Keywords:** microbial fuel cell, biosensor, *Pseudomonas aeruginosa*, silver nanoparticles

## Abstract

Simultaneous monitoring of antimicrobial responses to bacterial metabolic activity and biofilm formation is critical for efficient screening of new anti-biofilm drugs. A microbial fuel cell-based biosensor using *Pseudomonas aeruginosa* as an electricigen was constructed. The effects of silver nanoparticles (AgNPs) on the cellular metabolic activity and biofilm formation of *P. aeruginosa* in the biosensors were investigated and compared with the traditional biofilm detection method. The crystal violet staining results showed that the concentration of AgNPs being increased to 20 and 40 μg/mL had a slight and obvious inhibitory effect on biofilm formation, respectively. In comparison, the detection sensitivity of the biosensor was much higher. When the concentration of AgNPs was 5 μg/mL, the output voltage of the biosensor was suppressed, and the inhibition gradually increased with the AgNPs dose. AgNPs inhibited the activity of planktonic cells in the anolyte and the formation of biofilm on the anode surface, and it had a dose-dependent effect on the secretion of phenazine in the anolyte. The biosensor could monitor the impacts of AgNPs not only on biofilm formation but also on cell activity and metabolic activity. It provides a new and sensitive method for the screening of anti-biofilm drugs.

## 1. Introduction

Bacterial infection is a serious hazard to human health. The statistical research of NIH shows that more than 60% of infectious diseases are related to bacterial biofilms [1], such as periodontitis, diffuse bronchitis, pulmonary cystic fibrosis, valve endocarditis, and staphylococcus aureus chronic osteomyelitis [2]. In addition, the implantation of biomaterials and medical devices often fails due to biofilm infection [3]. A variety of bacteria can form biofilms, such as *Pseudomonas aeruginosa*, *Staphylococcus epidermidis*, *Escherichia coli*, *Klebsiella pneumonia*, *Proteus mirabilis*, *Streptococcus viridans*, *Streptococcus aureus*, and *Enterococcus faecalis* [1,4]. Antibiotics are the main method of clinical treatment of bacterial biofilm infection. However, the resistance of bacteria to antibiotics is greatly enhanced as biofilms are formed. This causes the recurrence of bacterial biofilm infections or even the failure of medical device implantation, which greatly increases the difficulty and cost of treatment [4]. The screening of new anti-biofilm drugs with new inhibition pathways or targets has become a hot issue [5]. As the traditional plate colony count method suffers from the limitation of being time-consuming, a high-throughput method based on 96-well plates is used to study pathogenic biofilm drug resistance [6]. However, the process of transferring biofilm can easily cause pollution and is tedious [7]. Currently, the widely used method is to measure the biomass or cell activity of the biofilm by colorimetry and fluorescent probe staining, such as crystal violet staining, Alamar Blue assay, and SYTO9 and PI staining [8,9,10,11,12]. Among them, the crystal violet staining method and Alamar Blue assay are endpoint detection methods. In particular, the crystal violet staining method used for biofilm quantification cannot distinguish between dead cells and live cells. SYTO9 and PI staining can detect bacterial vitality but these methods rely on laser confocal microscopy instruments. In addition, other techniques have been established to evaluate the metabolic activity of biofilm by labeling with isotopes or fluorescent substrates, such as adenosine triphosphate (ATP) bioluminescence [13] and Raman spectromicroscopy [14]. However, these methods require the labeling and destruction of cells. Recently, some studies have used label-free resonant hyperspectral imaging [15] as well as optoelectronic devices [16] to monitor bacterial attachment and antibiotic responses to early biofilm formation in real time. However, these label-free detection methods cannot distinguish between the bactericidal and bacteriostatic effects of antimicrobials. It is known that slow-growing or nongrowing cells in biofilms are very insensitive to many antimicrobials. Moreover, it has been demonstrated that the heterogeneity of cellular metabolic states in biofilms enables some cells to survive any metabolically directed attack and thus leads to biofilm resistance to antimicrobial agents [2]. Therefore, it is critical to develop advanced label-free monitoring techniques to screen antimicrobials that target the metabolic activities and biofilm formation of pathogens.

*P. aeruginosa* is an opportunistic pathogen that can easily develop multidrug resistance and form biofilms attacking human health [17]. Various nanoparticles have been exploited as anti-biofilm drugs with new inhibition mechanisms due to their intrinsic advantages of a high surface-area-to-volume ratio, increased reactivity, altered electronic properties, and magnetic properties. Such materials include metal/metal oxide nanoparticles, polymeric nanoparticles, nanoenzymes, nanocapsules, liposomes, and hydrogels [18]. Metal nanoparticles with the ability to target bacterial biofilm-related genes have attracted great interest as potent anti-biofilm agents [19]. Silver nanoparticles (AgNPs) are a well-known antimicrobial with broad-spectrum antibacterial, antifungal, and antiviral activity [20,21,22]. In particular, AgNPs with an average particle size of about 25 nm were found to effectively prevent the formation of *P. aeruginosa* biofilms [23]. Therefore, AgNPs with this particle size were chosen as anti-biofilm model drugs in this paper.

Microbial fuel cells (MFCs) with the ability to directly convert biological signals into electricity signals can be used as biosensors in the real-time monitoring of toxicity [24], biochemical oxygen demand [25], dissolved oxygen [26], and volatile fatty acids [27]. MFC-based biosensors continuously monitor the accumulated electrical outputs from the extracellular electron transfer in the presence of toxicity, and the accumulated electrons directly reflect the bacterial viability and metabolism. Therefore, MFC-based biosensors have been used for drug-sensitivity tests of *Staphylococcus aureus* and *Escherichia coli* to beta-lactam antibiotics [28], *Shewanella loihica* PV-4 to tobramycin, and mixed-culture biofilm from wastewater to tobramycin [29,30]. *P. aeruginosa* is a typical extracellular electrogenic strain, which can secrete a variety of phenazine compounds as electronic mediators to promote extracellular electron transfer between bacterial cells and electrodes in MFCs [31]. MFC-based biosensors with *P. aeruginosa* as an electricigen were developed for antibiotic susceptibility testing [32,33] and the detection of 3, 5-dichlorophenol in water [34]. In addition, MFC-based biosensors are proposed to have the potential to monitor cellular metabolic activity in biofilms [35]. However, there have been no reports of the use of MFC-based biosensors to study the response of antimicrobials to biofilm formation and bacterial metabolic activity in label-free biofilms. Real-time evaluation of metabolic activity in label-free biofilms is crucial and challenging for distinguishing the bactericidal and bacteriostatic effects of anti-biofilm drugs. In this study, we provide a novel method for simultaneous monitoring of the effects of anti-biofilm drugs on bacterial metabolic activity and biofilm formation. Briefly, an MFC-based biosensor with *P. aeruginosa* as an electricigen was constructed, and then the effects of AgNPs on the metabolic activity and biofilm formation of *P. aeruginosa* were monitored using MFC-based biosensors and further compared with the traditional biofilm detection method of crystal violet staining.

## 2. Materials and Methods

### 2.1. Preparation and Characterization of Silver Nanoparticles

Silver nanoparticles (1 mg/mL) with a diameter of 25 nm were purchased from Huzheng Nanotechnology Co., Ltd (Shanghai, China). Nanoparticles were dialyzed in deionized water for 48 h, sterilized through a 0.22 mm filter (Millipore, MA, USA), and kept at 4 °C for further use [36]. A transmission electron microscope (TEM, H-7650, HITACHI, Tokyo, Japan) was used to observe the morphology and structure of the nanoparticles. The particle size distribution and average particle size were calculated using the Nano Measurer software 1.2.5.

### 2.2. Cell Culture

*P. aeruginosa* strain (ATCC 9027) was cultivated in 10 mL Luria–Bertani (LB) broth (tryptone 10 g/L, yeast extract 5 g/L, and sodium chloride 10 g/L) at 37 °C, 150 rpm for 10 h. After centrifugation, cells were resuspended in the anolyte (Na_2_HPO_4_ 6 g L^−1^, KH_2_PO_4_ 3 g/L, NH_4_Cl 1.0 g/L, NaCl 0.5 g/L, MgSO_4_ 0.2 g/L, and CaCl_2_ 0.08 g/L) with glucose (10 g/L) and added into MFCs with a final OD_600_ value of 0.03 [37]. Alternatively, the harvested cells were resuspended in fresh LB medium and added into microwell plates with a final OD_600_ value of 0.03.

### 2.3. MFC-Based Biosensor Operation

A two-chamber MFC (125 mL of each chamber) separated by a proton exchange membrane (PEM, Nafion 117, Dupont, DE, USA) was used in this work [37]. Carbon brushes were used for the electrodes (both the diameter and the length of 3 cm). The *P. aeruginosa* cells and silver nanoparticles with a final concentration of 5 mg/mL, 10 mg/mL, 20 mg/mL, and 40 mg/mL were added into the anodic chamber filled with the anolyte with glucose. The cathodic chamber was filled with 50 mM K_3_[Fe(CN)_6_] in a 0.1 M phosphate buffer solution. The MFC was connected with a 1 KΩ external resistor and incubated at 37 °C for the power generation test. The output voltages of MFC-based biosensors were monitored continuously using a multi-channel data acquisition board (NI USB-6008, Beijing Huatai Oriental Technology Company, Beijing, China).

### 2.4. Characterization of Cell Activity and Metabolic Activity in MFC-Based Biosensors

A total of 2.0 mL of the anolyte was taken from an anodic chamber at different discharging times and centrifuged at 8000 rpm for 10 min. The supernatant was determined at 370 nm for evaluation of the phenazine concentration secreted by cells with a UV–Vis spectrophotometer (UV-1800, Mapada Instrument, Shanghai, China) [38]. The harvested cells were resuspended in 2 mL fresh LB medium and mixed well. In total, 100 μL of cell suspension and 10 μL of WST-1 solution were added into a 96-well plate and mixed well. After 2 h incubation, the cell activity was detected at 450 nm by a microplate reader (SpectraMax 250, Molecular Devices Corporation, Sunnyvale, CA, USA).

### 2.5. Protein Content of Biofilm on the Electrode Surface

The protein content was determined using a Bradford spectrophotometer [39]. After power generation, 0.5 g carbon brush fiber of the anode was cut off and soaked in 1 mol/L NaOH solution for 1 h. In total, 20 μL of sample solution and 200 μL of Coomassie Brilliant Blue G-250 were mixed and dyed for 2 min and then the absorbance was measured at 595 nm with a UV–Vis spectrophotometer [38]. The absorbance of the sample was compared with the standard curve to calculate its protein content.

### 2.6. Morphology Observation of the Biofilm

After power generation, the anode attached with biofilm was rinsed with PBS and immersed in a 2.5% glutaraldehyde solution for 4 h. Then, it was sequentially dehydrated with different concentrations of ethanol (10%, 30%, 50%, 70%, 80%, 95%, and 100%) for 10 min, followed by air-drying. The morphology of the biofilm on the anode was observed by scanning electron microscopy (SEM, SU8010, HITACHI). The morphology of the biofilm on the cover glass in a microwell plate was prepared in the same manner as for SEM observation.

### 2.7. Crystal Violet Staining

The recovered *P. aeruginosa* cells were centrifuged and resuspended in a fresh LB medium to adjust to the appropriate OD_600_ value. A total of 100 μL of cell suspension was co-cultured with 100 μL of fresh LB medium containing AgNPs with a final concentration of 5 mg/mL, 10 mg/mL, 20 mg/mL, and 40 mg/mL in a microwell plate at 37 °C for 24 h. The culture medium of each pore was removed, and each pore was rinsed with PBS solution and dried. Then, 250 μL of formaldehyde solution was added, the sample was fixed for 5 min, and the solution was pipetted out; 250 μL of 0.1% crystal violet solution was added, the sample was dyed for 20 min, and the solution was pipetted out, before rinsing with PBS solution to wash off the uncombined dye; 250 μL of 95% ethanol solution was added and retained for 5 min to elute the crystal violet dye in the cells; and 150 μL of ethanol eluent for each well was transferred into a new 96-well plate to measure the absorbance at 595 nm with the microplate reader [36].

### 2.8. Statistical Analysis

Data are presented as the mean ± standard deviation, and statistical differences between groups were determined using *t*-tests or a one-way analysis of variance. Significant difference values were indicated as follows: * *p* < 0.05, ** *p* < 0.01, and *** *p* < 0.001.

## 3. Results and Discussion

### 3.1. Morphology, Structure, and Size of Silver Nanoparticles

**Figure 1:**
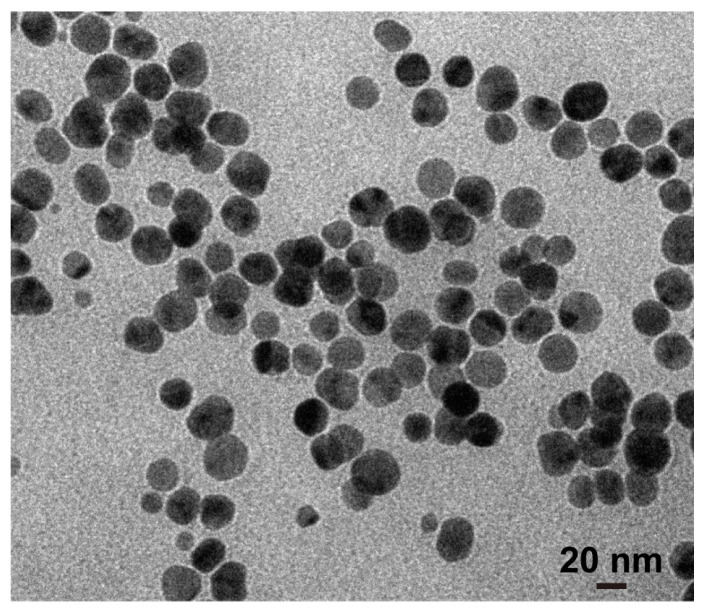
TEM image of silver nanoparticles.

The TEM diagram of silver nanoparticles is shown in Figure 1. Silver nanoparticles have a compact spherical structure, and the average particle size of silver nanoparticles is 25.70 ± 3.55 nm. The particle size distribution of silver nanoparticles is shown in Figure S1.

### 3.2. Effect of Silver Nanoparticles on the Biofilm Formation of P. aeruginosa in Microwell Plates by Crystal Violet Staining

**Figure 2:**
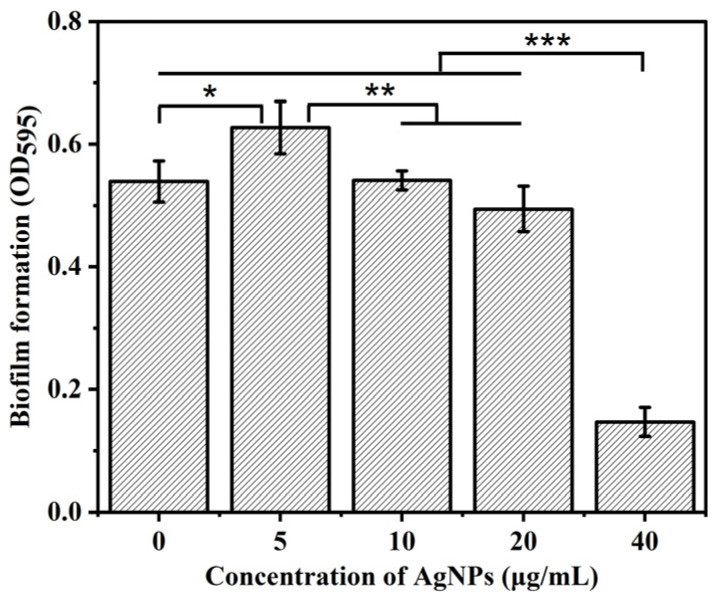
The effects of different concentrations of silver nanoparticles on *P. aeruginosa* biofilm formation by the crystal violet staining method (*n* = 4); * *p* < 0.05, ** *p* < 0.01, and *** *p* < 0.001.

The effect of AgNPs on the biofilm formation of *P. aeruginosa* was evaluated using the traditional crystal violet staining method on microwell plates. The crystal violet staining was used as an endpoint method. Therefore, the biofilms co-cultured with different concentrations of AgNPs after 24 h were evaluated. As shown in Figure 2, when the concentration of AgNPs was 5 μg/mL, the formation of *P. aeruginosa* biofilm was promoted by 16.7% compared to the control group. The minimal inhibitory concentrations of AgNPs for different *P. aeruginosa* strains varied over a large range, such as 0.4 μg/mL and 500 μg/mL for AgNPs (chemically synthesized, 20~25 nm) [40,41]. The synthesis method, size, shape, and stabilizing agents resulted in the different properties of AgNPs [42]. The promotion of biofilm formation by exposure to sublethal concentrations of AgNPs has already been reported [42,43,44]. A similar result showed that 4 μg/mL AgNPs promoted the increase in biofilm mass due to an increase in extracellular polymeric substances (EPSs) caused by a stress response to sublethal silver concentrations [42]. The upregulation of quorum sensing, EPS production, and antibiotic resistance genes associated with bacterial exposure to sublethal AgNP concentrations resulted in the stimulation of biofilm formation [44]. However, when the concentration of AgNPs was 10–40 μg/mL, the inhibitory effect of AgNPs on the biofilm of *P. aeruginosa* gradually increased with the AgNP concentration. When the concentration of AgNPs was 40 μg/mL, the inhibitory effect on the biofilm formation of *P. aeruginosa* was the most significant, and the inhibition ratio was 72.7%, which confirmed the strong antibacterial effect of AgNPs. It was reported that AgNPs could enter cells, inactivating cellular enzymes and generating endogenous ROS by interaction with biomolecules [19,20,45]. With the increase in AgNP concentration, the number of cells decreased, leading to the reduction in biofilm formation.

**Figure 3:**
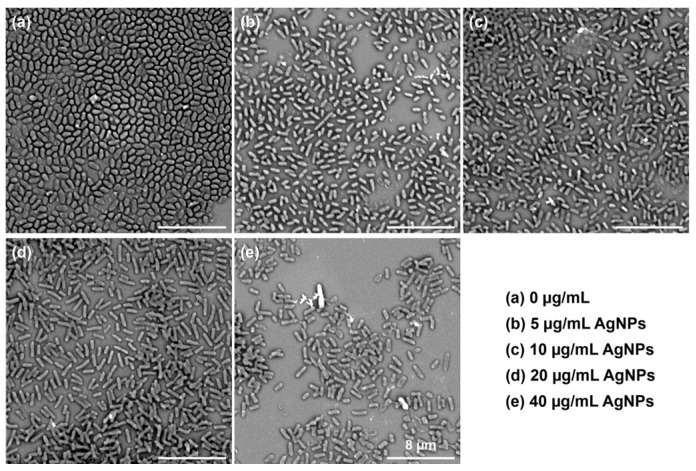
SEM images of *P. aeruginosa* biofilms on the cover glasses co-cultured with different concentrations of silver nanoparticles for 24 h.

The SEM images of *P. aeruginosa* biofilms on the cover glasses co-cultured with different concentrations of AgNPs in microwell plates after 24 h are shown in Figure 3. A thick biofilm with closely arranged cells in a plump and short rod shape was formed on the surface of the cover glass without the addition of AgNPs. With the increase in AgNP concentration, the number of cells in the biofilm decreased, and the distance between them increased. The shape of the cells also became slim and elongated. This was the most obvious at the concentration of 40 μg/mL AgNPs. These results verified the anti-biofilm activity of AgNPs. Interestingly, AgNPs at a concentration of 5 μg/mL exhibited an obvious inhibition of biofilm formation, which is different from the crystal violet staining results. This suggested that there were some slight differences among different endpoint detection methods. Therefore, it is urgent to develop accurate screening methods for anti-biofilm agents.

### 3.3. Effect of Silver Nanoparticles on the Output Voltage of MFC-Based Biosensors

**Figure 4:**
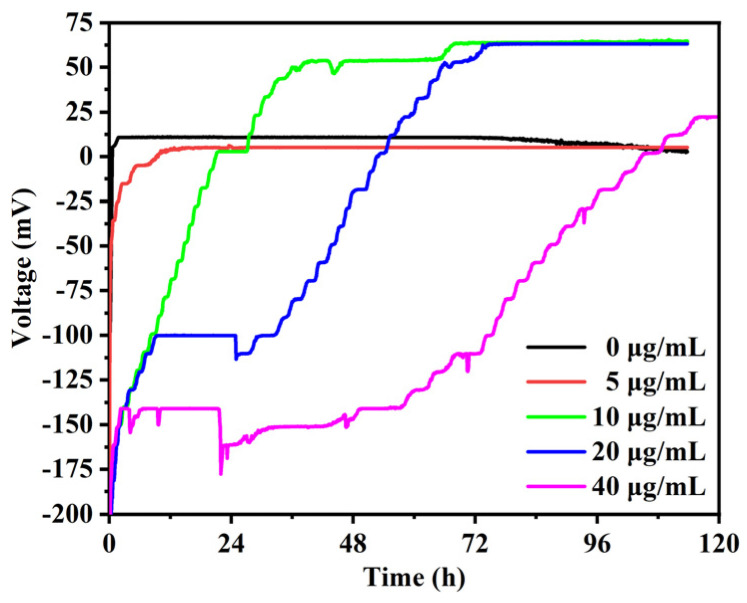
The output voltage curves of MFC-based biosensors with *P. aeruginosa* co-cultured with different concentrations of silver nanoparticles.

A new screening method was constructed for an MFC-based biosensor to assess the anti-biofilm activity of AgNPs against *P. aeruginosa* biofilms. Compared with the crystal violet staining method in microwell plates, MFC-based biosensors could monitor the interaction between *P. aeruginosa* biofilms and AgNPs by an electrical signal in real time without labeling. As shown in Figure 4, the biosensor without the addition of AgNPs could generate an output voltage in ~0.6 h and then maintain a stable output voltage of ~11.0 mV for 73 h. The output voltage was much lower than the value of ~210.0 mV reported by Qiao et al. [37]. This difference in output voltage might be attributed to the fact that the number of cells inoculated in our biosensors was much lower than that in the literature (OD_600_ of 0.03 versus OD_600_ of 1.0). After the addition of AgNPs, the biosensors exhibited different output voltage curves with a negative starting voltage in a long start-up time and a delayed recovery voltage. The AgNPs obviously inhibited the starting voltage and the start-up time of biosensors, and the inhibition was increased with AgNP concentrations. The MFC-based biosensors, with the addition of 5 μg/mL AgNPs, generated a voltage after ~9.7 h and then maintained the voltage of 5.2 mV. However, after the addition of 10 μg/mL, 20 μg/mL, and 40 μg/mL AgNPs, the output voltage was significantly inhibited in ~21.0 h, ~53.0 h, and ~104.7 h, and then gradually generated a considerably high and stable voltage of 65.0 mV, 63.0 mV, and 22.2 mV, respectively. This suggested that AgNPs could suppress the electricity production of *P. aeruginosa* for a certain period and then result in a higher voltage. In addition, the MFC-based biosensors produced no output voltage after increasing the concentration of AgNPs to 80 μg/mL.

**Figure 5:**
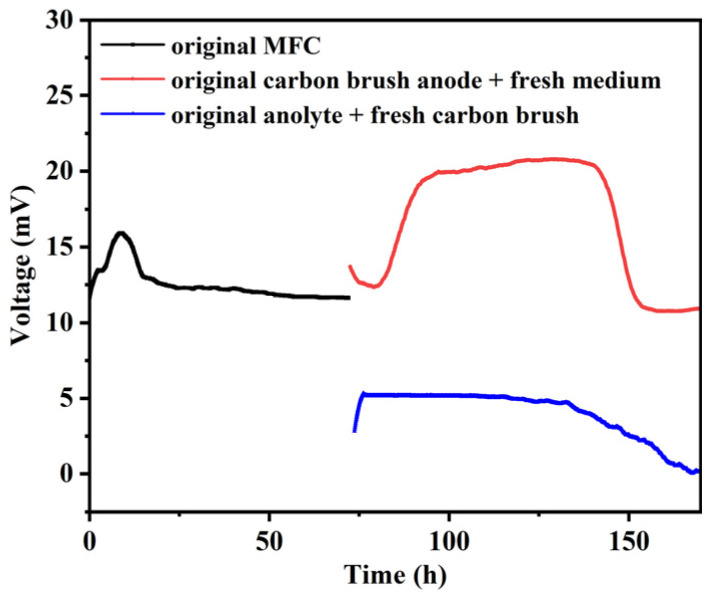
The discharge curves of the original MFC, the MFC containing the original carbon brush anode in a fresh medium, and the MFC containing the original anolyte with a fresh carbon brush.

To further investigate the main factors affecting the electricity production of *P. aeruginosa*, we explored the discharging curves of the original MFC-based biosensor, the MFC-based biosensor containing the original carbon brush anode in fresh medium, and the MFC-based biosensor containing the original anolyte with a fresh carbon brush. As shown in Figure 5, the original MFC-based biosensor generated a stable voltage of ~11.7 mV. After replacing the original medium with fresh medium, the biosensor with the original carbon brush in fresh medium gradually generated a higher voltage of ~20.0 mV. This was maintained for about 55 h, while the biosensor with a new carbon brush in the original medium generated a lower voltage of ~5.1 mV for about 40 h. These results demonstrated that a stable biofilm was formed on the anode surface of the MFC, which played an important role in generating a stable output voltage for the MFC. In addition, suspended cells and secreted electron mediators in the original electrolyte could contribute to the generation of stable output voltage. Therefore, the effects of AgNPs on the cell activity, metabolic activity, and biofilm formation of *P. aeruginosa* in MFC-based biosensors were further studied.

### 3.4. Effect of Silver Nanoparticles on the Cell Activity and Metabolic Activity of P. aeruginosa in MFC-Based Biosensors

**Figure 6:**
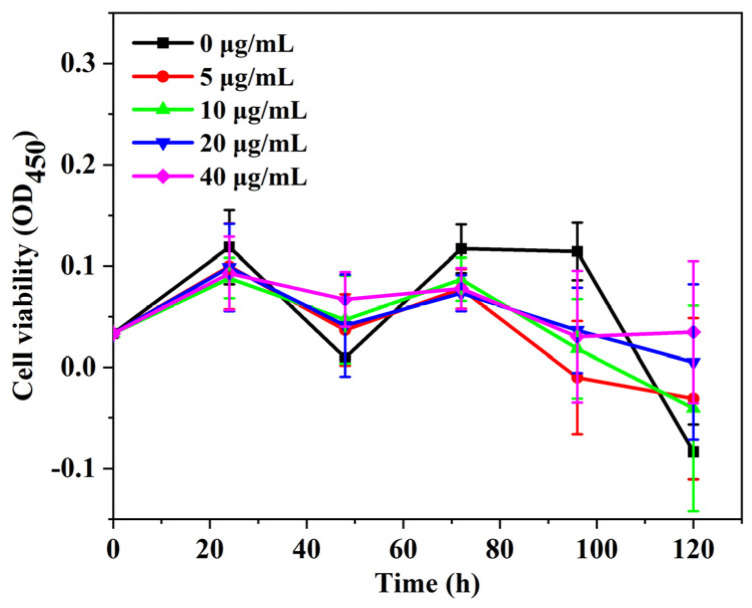
The cell activity of the planktonic cells co-cultured with different concentrations of silver nanoparticles in the anodic chamber of MFC-based biosensors (*n* = 6).

The effect of AgNPs on the cell activity of *P. aeruginosa* in MFC-based biosensors was investigated. Water-soluble tetrazolium (WST) can be reduced by dehydrogenase to orange-yellow water-soluble formaldehyde in the presence of electron coupling reagents, and the amount of formaldehyde generated is proportional to the number of living cells [46]. The cell activity of the planktonic cells in the anodic chamber of MFC-based biosensors was monitored at different discharging times by WST. As shown in Figure 6, the planktonic cells in MFC-based biosensors co-cultured with AgNPs exhibited similar activity-change curves but lower values compared to those cells without the addition of AgNPs. This verified that AgNPs inhibited the cell activity of planktonic cells in the anolyte. Furthermore, the value of the suspended cell activity was not constant but changed over time. The cell activity of suspension cells with AgNPs slightly increased within 24 h and then gradually decreased; subsequently, almost all planktonic cells in the anolyte lost their activity after 96 h. It was interesting that the cell activity of planktonic cells without the addition of AgNPs showed the most obvious minimum at 48 h and then gradually recovered until 96 h. The suspension cells lost their activity after 110 h. The strong minimum could be attributed to the massive consumption of substrates by the rapid growth of cells in the anolyte, accompanied by the inhibition of cell activity and cell death. The surviving cells continued to grow and metabolize; thus, the cell activity gradually increased and then decreased until the substrates were completely depleted.

**Figure 7:**
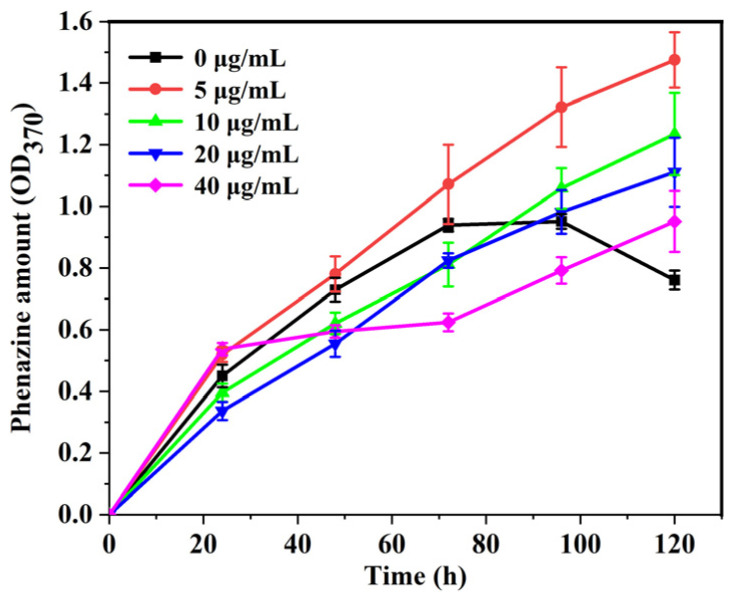
The amount of phenazine secreted by cells co-cultured with different concentrations of silver nanoparticles in the anodic chamber of MFC-based biosensors (*n* = 3).

The effects of AgNPs on the cellular metabolic activity of *P. aeruginosa* in MFC-based biosensors were also investigated. Phenazine is an extracellular electron-transfer mediator secreted by *P. aeruginosa* cells that plays an important role in the electricity production efficiency of cells [31]. It was reported that the absorption peak at 370 nm can be used to evaluate the concentration of phenazine [38]. Therefore, the amount of phenazine secreted by cells co-cultured with different concentrations of AgNPs in the anodic chamber of MFC-based biosensors was monitored at 370 nm during the discharging time. As shown in Figure 7, the concentration of phenazine secreted by *P. aeruginosa* cells without the addition of AgNPs was increased in the early stage and then decreased in the later stage. In contrast, the amount of phenazine secreted by cells co-cultured with AgNPs continuously increased over 120 h and exceeded the blank control value at the later stage. As the concentration of AgNPs increased, the secreted phenazine concentration first increased and then gradually decreased. The cells co-cultured with 5 µg/mL AgNPs produced the highest concentration of phenazine.

### 3.5. Effect of Silver Nanoparticles on the Biofilm Formation of P. aeruginosa on the Anodes of MFC-Based Biosensors

**Figure 8:**
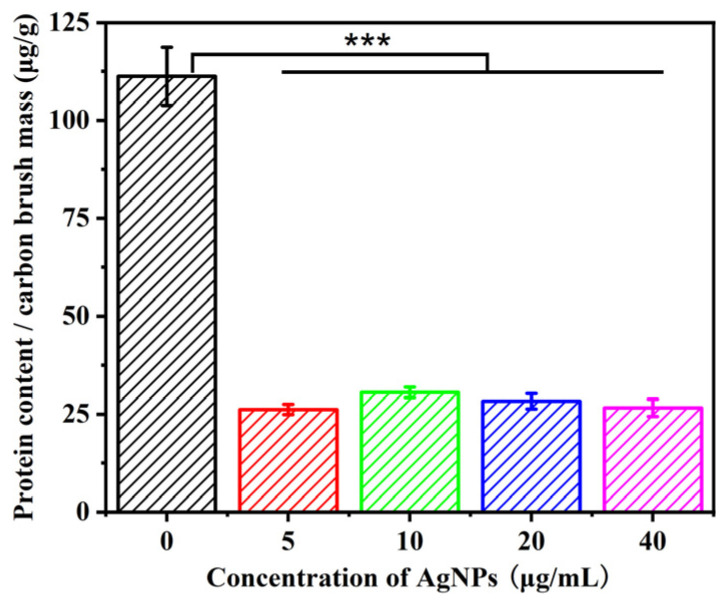
The protein content of biofilms attached to carbon brush anodes with different concentrations of silver nanoparticles in the anodic chamber of MFC-based biosensors (*n* = 3); *** *p* < 0.001.

**Figure 9:**
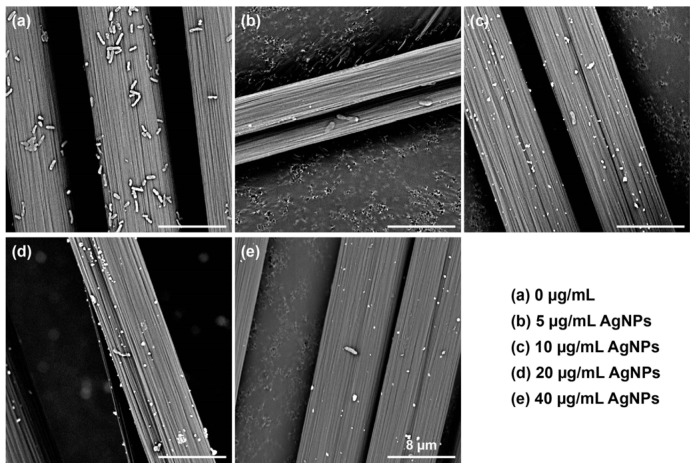
SEM images of *P. aeruginosa* biofilms on the anodes of MFC-based biosensors added with different concentrations of silver nanoparticles after 120 h.

The *P. aeruginosa* biofilms formed on the anodes of the biosensors were further assessed. As shown in Figure 8, the protein content of the biofilm attached to the surface of the carbon brush anode was significantly decreased with the addition of AgNPs, and no obvious difference between different AgNP concentrations was observed. The SEM results of the biofilms attached to the anodes were in accordance with the results for the determination of protein content. As shown in Figure 9, the amount and morphology of the biofilms formed on the anodes of the biosensors were significantly different from those of biofilms formed on the cover glasses in microwell plates. The cells attached to the electrode surface with or without AgNPs were all slim and elongated in shape. The addition of AgNPs only affected the number of attached cells on the electrode. When AgNPs were not present in the biosensor, a thin biofilm formed on the electrode surface. In contrast, when AgNPs were added to the biosensor, no biofilm formed on the surface of the electrode except for single cells. It suggested that AgNPs inhibited the biofilm formation on the anode. These results were in accordance with the cell activity results of planktonic cells. According to the experimental results of factors affecting electricity production, biofilms, suspended cell activity, and the secreted phenazine amount in the anolyte contributed to electricity signal generation. These results demonstrated that the suppressed electrical signals of MFC-based biosensors with the addition of AgNPs were indicative of the inhibition of the cell activity and biofilm formation of *P. aeruginosa* by AgNPs. In other words, MFC-based biosensors could monitor the impacts of AgNPs not only on biofilm formation but also on suspended cell activity.

Moreover, it was observed that the recovery voltage of the biosensor was progressively delayed with increases in AgNP concentration (Figure 4). In line with this, the amount of phenazine secretion in the biosensor gradually decreased with the increase in AgNP concentration, but in the later stage of power generation, the accumulated amounts of phenazine in all AgNP treatment groups were higher than that in the blank control group (Figure 7). Therefore, the accumulation of a high amount of secreted phenazine and the adaptation of bacteria to AgNPs might be the reasons for the delayed recovery voltage. In addition, it was reported that the phenazine secreted by cells could be adsorbed on the surface of the biofilm and lead to a low concentration of phenazine in the anolyte [37,38]. The decrease in phenazine concentration in the anolyte after 72 h without the addition of AgNPs (Figure 7) may be due to the adsorption of phenazine by the formed biofilm. After the addition of AgNPs, the formation of biofilms on the anodes was inhibited, and the secreted phenazine could not be adsorbed, thus resulting in an increase in phenazine concentration in the anolyte. Furthermore, with the increase in AgNP concentration, the inhibitory effect of AgNPs on bacterial metabolic activity was enhanced, resulting in a decrease in the concentration of phenazine secreted in the anolyte. Overall, the above results demonstrate that MFC-based biosensors can simultaneously monitor the effects of anti-biofilm drugs on cellular metabolic activity and biofilm formation.

The anti-biofilm results of AgNPs against *P. aeruginosa* cells in microwell plates and biosensors were compared. MFC-based biosensors have a higher detection sensitivity for AgNPs than crystal violet staining. For the crystal violet staining results, the concentration of 5 μg/mL AgNPs promoted the formation of *P. aeruginosa* biofilm after 24 h. Still, the inhibition was more pronounced as the concentration of AgNPs increased from 20 μg/mL to 40 μg/mL. For the MFC-based biosensor results, the starting voltage and the start-up time of biosensors were obviously inhibited by the concentration of 5 μg/mL AgNPs, and the inhibition increased with the AgNP concentration. The output voltage generated by MFC-based biosensors is a real-time indicator of cellular metabolic activity and biofilm status. Once AgNPs affect bacterial viability, cellular metabolic activity, or biofilm formation, extracellular electron transport is affected. Then, the output voltage of the MFC-based biosensor is immediately altered. However, crystal violet staining shows biofilm formation after 24 h of AgNP treatment. Therefore, the MFC-based biosensor has a higher detection sensitivity than the crystal violet staining method. Moreover, the MFC-based biosensor could monitor the impact of AgNPs on the cell activity, metabolic activity, and biofilm formation of *P. aeruginosa* in real time without labeling. The suppressed output voltage of the biosensor with the addition of AgNPs was ascribed to the inhibition of the cell activity of planktonic cells in the anolyte and the biofilm formed on the anodes. The subsequent recovery voltage could be due to the accumulation of phenazine secreted in the anolyte and the adaptation of bacteria to AgNPs.

## 4. Conclusions

An MFC-based biosensor with *P. aeruginosa* as an electricigen was constructed for the simultaneous monitoring of AgNPs’ impacts on the cell metabolic activity and biofilm formation of *P. aeruginosa*. The MFC-based biosensor results were also compared with crystal violet staining results. The biofilm formed on the glass covers in microwell plates by crystal violet staining was promoted by 5 μg/mL AgNPs. When the concentration of AgNPs increased from 20 μg/mL to 40 μg/mL, the inhibition of biofilm was more pronounced. In comparison, the MFC-based biosensor was more sensitive in detecting the inhibition of AgNPs. The output voltage of the biosensor was obviously inhibited by 5 μg/mL AgNPs, and the inhibition was increased with the AgNP concentration. The MFC-based biosensor could monitor not only the AgNPs’ impacts on the biofilm formation of *P. aeruginosa* but also the cell activity of planktonic cells in anolytes and their cellular metabolic activity. Therefore, MFC-based biosensors are promising for the screening of anti-biofilm drugs and have the potential to simultaneously monitor antimicrobial responses to bacterial metabolic activity and biofilm formation.

## Data Availability

Data are contained within the article.

## Supplementary Materials

The following supporting information can be downloaded at: https://www.mdpi.com/article/10.3390/bios14120606/s1, Figure S1: The particle size distribution of silver nanoparticles.
