# Supplementary material for: Antimicrobial Responses to Bacterial Metabolic Activity and Biofilm Formation Studied Using Microbial Fuel Cell-Based Biosensors
Source: Biosensors (Basel). 2024 Dec 11;14(12):606. doi: 10.3390/bios14120606 (PMC11674809; doi:10.3390/bios14120606)
Supplement: Supplementary file 1 [file biosensors-14-00606-s001.zip › biosensors-3257994-supplementary.pdf]

Antimicrobial Responses to Bacterial Metabolic Activity and Biofilm Formation Studied Using Microbial Fuel Cell-Based Biosensors

Wenguo Wu [1,*,†], Huiya Hong [1,†], Jia Lin [1] and Dayun Yang [2,*]

[1] College of Chemical Engineering, Huaqiao University, Xiamen 361021, China;

[2] Fujian Key Laboratory of Translational Research in Cancer and Neurodegenerative Diseases, School of Basic Medical Sciences, Fujian Medical University, Fuzhou 350108, China

[*] Correspondence: wuwenguo@hqu.edu.cn (W.W.); dyyang@fjmu.edu.cn (D.Y.)

[†] These authors contributed equally to this work.

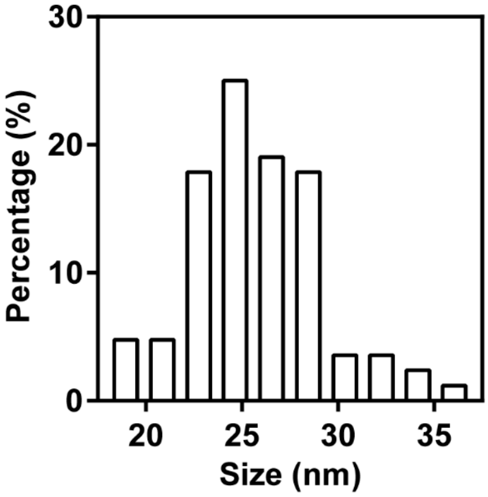

Figure S1. The particle size distribution of silver nanoparticles.
